# TR4 worsen urosepsis by regulating GSDMD

**DOI:** 10.1186/s40001-024-01742-6

**Published:** 2024-03-01

**Authors:** Huan Wang, Shibin Zhu, Zhenwei Zhou, Zhenghui Wang, Wei Zhuang, Dingwei Xue, Zeyi Lu, Qiming Zheng, Lifeng Ding, Liangliang Ren, Wenqing Luo, Ruyue Wang, Guangju Ge, Liqun Xia, Gonghui Li, Haiyang Wu

**Affiliations:** 1 Department of Urology, Sir Run Run Shaw Hospital, Zhejiang University School of Medicine, Hangzhou, 310016 China; 2 Department of Urology, The Second Affiliated Hospital of Fujian Medical University, Quanzhou, 362000 China

**Keywords:** TR4, Urosepsis, Pyroptosis, GSDMD

## Abstract

**Background:**

Urosepsis is a life-threatening organ disease in which pathogenic microorganisms in the urine enter the blood through the vessels, causing an imbalance in the immune response to infection. The aim of this study was to elucidate the role of testicular orphan receptor 4 (TR4) in urosepsis.

**Methods:**

The role of TR4 in the progression and prognosis of urosepsis was confirmed by analyzing data from online databases and clinical human samples. To mimic urosepsis, we injected *E. coli* bacteria into the renal pelvis of mice to create a urosepsis model. Hematoxylin and eosin staining was used to observe histopathological changes in urosepsis. The effects of the upregulation or downregulation of TR4 on macrophage pyroptosis were verified in vitro. Chromatin immunoprecipitation assay was used to verify the effect of TR4 on Gasdermin D (GSDMD) transcription.

**Results:**

TR4 was more highly expressed in the nonsurviving group than in the surviving group. Furthermore, overexpressing TR4 promoted inflammatory cytokine expression, and knocking down TR4 attenuated inflammatory cytokine expression. Mechanistically, TR4 promoted pyroptosis by regulating the expression of GSDMD in urosepsis. Furthermore, we also found that TR4 knockdown protected mice from urosepsis induced by the *E. coli*.

**Conclusions:**

TR4 functions as a key regulator of urosepsis by mediating pyroptosis, which regulates GSDMD expression. Targeting TR4 may be a potential strategy for urosepsis treatment.

## Introduction

Sepsis is a life-threatening systemic organ dysfunction syndrome (multiple organ dysfunction syndrome, MODS) caused by a dysregulated host response to infection [1, 2]. Approximately 19 million people worldwide are threatened by sepsis every year [3]. The overall case fatality rate is as high as 25% [4], and the prognosis of patients is poor; therefore, these patients should receive increased attention [5, 6]. Urosepsis is caused by urogenital infection and accounts for approximately 9% of sepsis cases [3]. The main reason is that obstruction of the urinary tract causes high pressure in the renal pelvis, and the surface toxins of urine-derived bacteria enter the blood, further causing systemic sepsis [7]. Gram-negative bacilli are the main causative organisms of urinary sepsis, of which *E. coli* is the most common. The current understanding of the key mechanisms regulating the host response to sepsis and its progression to organ dysfunction, coupled with the multifactorial nature of sepsis etiology, has limited therapeutic options for urosepsis.

One of the most important pathophysiologic features of urosepsis is an imbalance of cytokines [8]. Cytokines, such as interleukin 2 (IL-2), interleukin 6 (IL-6), interleukin 8 (IL-8), and tumor necrosis factor α (TNF-α), cause neutrophil–endothelial cell adhesion, activate complement and coagulation cascades, and lead to the production of microthrombi [9–11]. Typically, sepsis is an overwhelming response to infection followed by an immunosuppressive phase characterized by allergy, reduced lymphocytes, and secondary infections. The intensity of immune responses depends on a variety of factors, including host genetics and the pathogen features [12, 13]. However, mortality in early sepsis is attributable to an acute, systemic proinflammatory response. Notably, controlling cytokine storms has been proven to improve the prognosis of patients with sepsis [14, 15]. The mechanisms that cause cytokine storms may be key to future powerful strategies for addressing sepsis, but they remain largely unknown.

Pyroptosis is a newly discovered programmed cell death process linked to inflammation. It is characterized by apoptosis and necrosis in both macrophages and nonmacrophage cells [16, 17]. Pyroptosis is characterized by the formation of pores in the plasma membrane, cell swelling and membrane rupture, resulting in massive leakage of cell cytokines. The Gasdermin (GSDMS) family comprises the key molecules that form pores in the plasma membrane [18]. GSDMs are cleaved by the caspase-1-induced canonical pathway or caspase-11/4/5-induced noncanonical pathway [19, 20]. Mounting evidence indicates that pyroptosis plays a key role in sepsis [17]. Although moderate pyroptosis in sepsis can control infection, overactivated pyroptosis can trigger dysregulation of the host immune response and even the septic shock [21]. Shao group revealed that lipopolysaccharide (LPS) can directly bind to caspase-11/4/5 to trigger pyroptosis [22]. In addition, N-terminal GSDMD fragments trigger macrophages pyroptosis via a phospholipase C Gamma 1 (PLCG1)-dependent mechanism.

Testicular orphan receptor 4 (TR4, also named NR2C2), a member of the nuclear receptor family, plays a critical role as a transcription factor in various biological processes, including neuronal and bone development, protecting cells from oxidative stress [23–25]. Importantly, TR4 also plays a critical role in macrophage-associated foam cell formation in cardiovascular diseases [26] and *Mycobacterium tuberculosis* disease [27]. Previous studies from our laboratory have shown that TR4 suppressed macrophage infiltration via alteration of TIMP-1/MMP2/MMP9 signaling [28].These studies indicated that there are close connections between TR4 and the function of macrophages. Recently, Li et al. reported that TR4 was highly expressed in the testes and that the expression of TR4 was upregulated in testicular macrophages in an LPS-induced mouse orchitis model in *vivo*. Mechanistically, TR4 promoted the expression of IL-1β and IL-6 by activating NF-κB signaling [29]. However, the link between TR4 and its influence on macrophages in urosepsis remains unclear and requires further clarification.

**Fig. 1:**
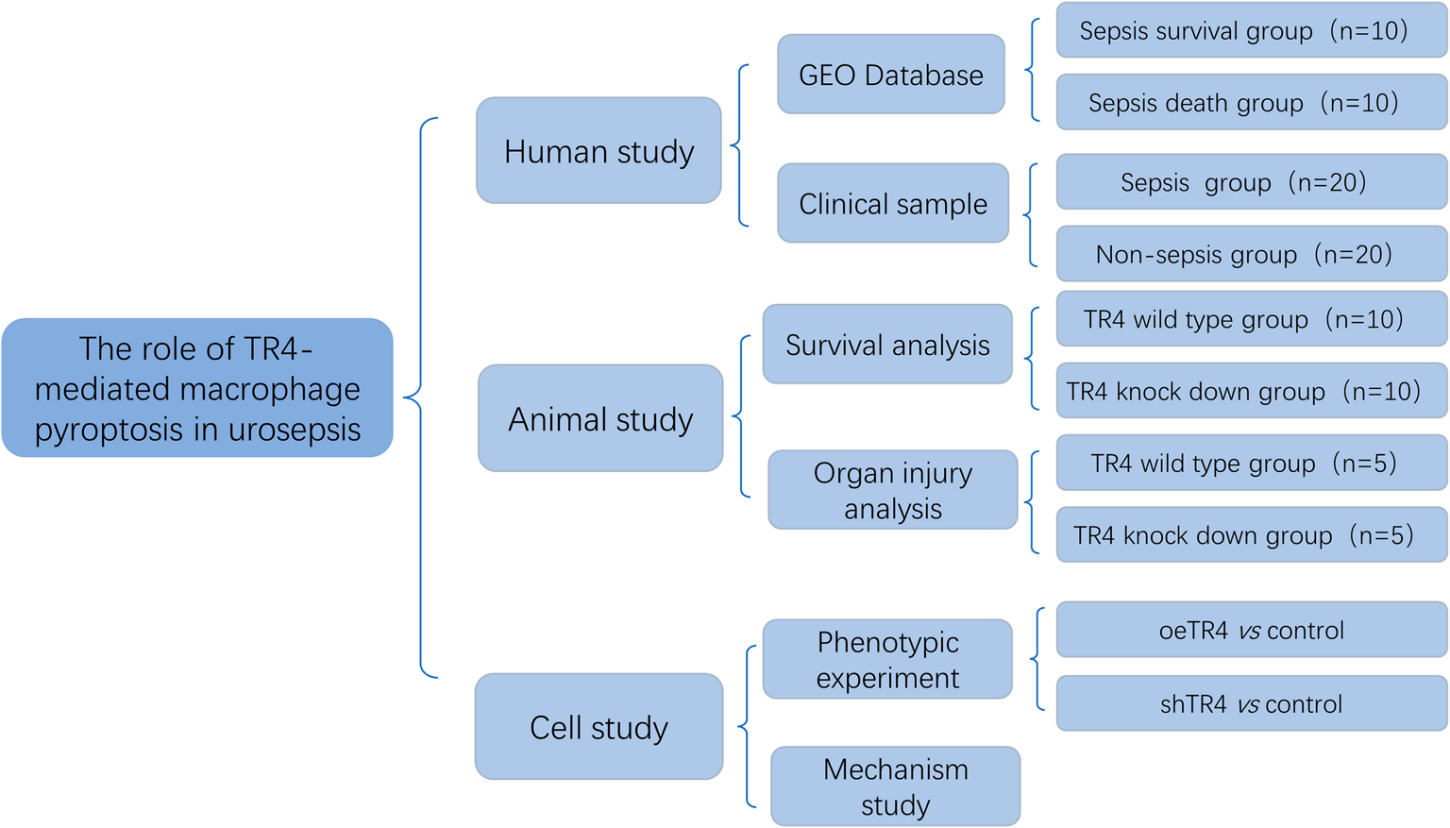
Workflow

The present study aimed to clarify the effect of TR4 in urosepsis and investigate the potential mechanism through which TR4 is involved in urosepsis via GSDMD-induced pyroptosis. Figure 1 shows the workflow of this study.

## Methods

### Clinical samples

Clinical blood samples were obtained from patients who were diagnosed with urinary stones at the Sir Run Run Shaw Hospital of Zhejiang University. Patients were divided into sepsis and nonsepsis groups according to whether sepsis occurred after lithotripsy surgery. All samples were collected with informed consent according to the Internal Review and the Ethics Board of Sir Run Run Shaw Hospital.

### In vivo animal studies

The transgenic mice were characterized previously [30]. In brief, TR4-knockdown mice (TR4^±^) were generated at the Nanjing Biomedical Research Institute of Nanjing University. Transgene integration was confirmed by genomic PCR and western blotting. Six-week-old transgenic mice or WT mice were subjected to urosepsis by injecting *E. coli* bacteria (2.5 × 10^9^/mL) into the kidney after which the ureter was blocked. Mortality was assessed every 6 h. Furthermore, H&E staining of the kidney, lung, liver, and intestine was performed to analyze the degree of the damage.

### Histopathological evaluation

H&E staining was performed according to the manufacturer’s instructions. First, mouse tissues were fixed in 10% formalin for 48 h. Then, the tissues were embedded in paraffin and cut into 5 μm thick sections. The slices were subjected to gradient dehydration and rehydration and were then stained with hematoxylin and eosin. Sepsis progression was assessed through observation of organ damage and inflammatory cell infiltration under a microscope. We assessed and scored histological lung injury [31], liver injury [32], kidney injury [33], and intestinal injury [34] using reported criteria. Briefly, according to the number of red blood cells and inflammatory cells, the injury score was divided into 5 levels: 1, minimal damage;2, mild damage; 3, moderate damage; 4, severe damage; and 5, maximal damage.

### Cell culture

The human leukemia monocytic cell line THP-1 was obtained from the American Type Culture Collection (ATCC, Manassas, VA, USA) and cultured in RPMI-1640 media supplemented with 10% FBS (Gibco, Thermo Fisher Scientific, USA), 0.05 mM β-mercaptoethanol, penicillin and streptomycin at 37 °C in a 5% CO_2_ atmosphere. The medium was changed every three days. Certification of the THP-1 cell lines was performed in the last three years using the STR assay. M0 cells were induced from the THP-1 cells by culture with PMA (100 ng/mL) for 48 h. M1 cells were induced from the M0 by culture with LPS (100 ng/mL) for 48 h.

### Western blot analysis

Western blotting was performed as described in our previous study [35]. Briefly, cells were harvested and washed three times with cold PBS. Then, the cells were lysed in RIPA buffer supplemented with protease inhibitor cocktails (Millipore, Billerica, MA, USA). The proteins were boiled at 100 ℃ for 20 min and subsequently stored at − 20 ℃ until analysis. Equal amounts of proteins were separated by 10% SDS–PAGE. Then, the proteins were transferred onto PVDF membranes (Thermo Fisher Scientific, USA). After blocking with nonfat milk on the incubator shaker for 1 h at room temperature, the membranes were incubated with primary antibodies at 4 °C overnight, which included GAPDH (1:1000, sc-202525, Santa Cruz, CA, USA), GSDMD (1:1000, A18281, ABclonal, Wuhan, China), and TR4 (1:1000, ab109301, Abcam, USA). On the second day, the membranes were hybridized with a secondary antibody of mouse or rabbit at room temperature for 1 h. The immunoreactive signals were visualized by an enhanced chemiluminescence kit (FD8000, FUDE Biological Technology CO, Ltd, Hangzhou, China).

### siRNA and plasmid transfection

For siRNA transfection, THP-1 cells (5 × 10^4^/well) were planted in a 6-well culture plate. When the cells reached approximately 40–50% confluence, they were transfected with siTR4 (CGGGAGAAACCAAGCAA) or the negative control (purchased from RiboBio, Guangzhou, China) using RFect siRNA/miRNA Transfection Reagent (Baidai, Changzhou, China) according to the manufacturer’s instructions. After incubating for 48 h, the cells were collected for further study.

For plasmid transfection, Lipofectamine 3000 (Invitrogen, Thermo Fisher Scientific, USA) was used to transfect plasmids containing full-length TR4 cDNA fragments designed and synthesized by GeneChem (Shanghai, China) according to the manufacturer’s instruction. After transfecting for 8 h, the culture medium was changed. The transfected cells were incubated for 48 h.

### RNA extraction and quantitative real-time PCR

Total RNA from cells or tissue was isolated using an RNA-Quick Purification Kit (Yeasen Biotech, Shanghai, China), and the concentration was determined using a Nanodrop 2000 (Thermo Fisher, USA). Reverse transcription was performed using EasyQuick RT MasterMix (CWBIO, Beijing, China), and qRT-PCR was performed using SYBR Green PCR Master Mix (CWBIO, Beijing, China) in a LightCycler 480 System (Roche, Germany). Specific mRNA concentrations were analyzed using the 2 − ▵▵Ct method. GAPDH was used as the normalization control. The primers used were synthesized by Tsingke Biological Technology (Beijing, China). TR4 primer sequence, forward: 5′‐GGCTCTGAACCTGCCTCTG‐3′; reverse: 5′‐AGGATGAACTGCTGTTTGGG‐3′. GAPDH primer sequence, forward: 5′‐GGAGTCAACGGATTTGGT‐3′; reverse: 5′‐GTGATGGGATTTCCATTGAT‐3′. GSDMD primer sequence, forward: 5′-ATGAGGTGCCTCCACAACTTCC‐3′; reverse: 5′-CCAGTTCCTTGGAGATGGTCTC‐3′. IL-1β primer sequence, forward: 5′-TCCAGGGACAGGATATGGAG‐3′; reverse: 5′-TCTTTCAACACGCAGGACAG‐3′. MCP1 primer sequence, forward: 5′-AGAATCACCAGCAGCAAGTGTCC-3′; reverse: 5′-TCCTGAACCCACTTCTGCTTGG-3′. IL-6 primer sequence, forward: 5′-AGACAGCCACTCACCTCTTCAG-3′; reverse: 5′-TTCTGCCAGTGCCTCTTTGCTG-3′. TNF-α primer sequence, forward: 5′-CTCTTCTGCCTGCTGCACTTTG-3′; reverse: 5′-ATGGGCTACAGGCTTGTCACTC-3′.

### IL-1β measurement

The IL-1β concentration in the supernatant of the cells was measured via an IL-β ELISA kit (Beyotime, China) according to the manufacturer’s instructions. An automated microplate reader (Thermo Fisher Scientific, USA) was used for the measurement of the optical density (OD) at 450 nm. The concentrations of each sample were determined based on the optical density and the concentration of the standard sample.

### PI/Hoechst staining

THP-1 cells transfected with the TR4 plasmid or siRNA were planted in 24-well plates. First, the cells were cultured overnight with PMA (100 ng/ml) and stimulated with 500 ng/mL LPS for 4 h. Finally, 2 μg/mL PI and 5 μg/mL Hoechst were added, and the cells were incubated at room temperature for 10 min. Afterwards, the cells were observed with the fluorescence microscope, after which the proportion of PI-positive cells among the total cells was determined.

### ChIP assay

ChIP assays were conducted using the SimpleChIP® Enzymatic Chromatin IP Kit (CST, Massachusetts, United States) according to the manufacturer’s instructions. Briefly, 293 T cells (1 × 10^7^) were collected and cross-linked with 1% formaldehyde for 10 min at room temperature. Then, the genomic DNA was cleaved into fragments ranging from 200 to 500 bp by sonication. 2% of the solution was used to assess the input complex, and the remaining lysates were immunoprecipitated with a TR4 antibody (ab109301; Abcam, United States) at 4 °C overnight. IgG was used as a control. The DNA from the input or immunoprecipitated samples was extracted and analyzed via qRT-PCR. The products were separated via 2% agarose gel electrophoresis. We designed specific primers to identify the target sequence in the human GSDMD promoter, as follows: F, 5′-GAACTGAGTGTGGACAGAGCA-3′; R, 5′-TCAACGGGCAGTGACGAAG-3′.

### Survival analysis with GEO data

The TR4 expression data and clinical information of patients with sepsis were collected from the GEO database (GSE48080) [36]. A total of 20 patients who were diagnosed with sepsis were included in this study to investigate the association between TR4 expression and the prognosis of sepsis. The differential expression gene analyses were performed using the ACLBI tool (https://www.aclbi.com/) [37].

### Statistical analysis

All the statistical analyses were performed using the GraphPad Prism 8 (GraphPad Software, Inc, La Jolla, CA, United States). Each experiment was repeated at least three times. All the data are presented as the means ± SDs. The differences between the experimental groups were evaluated by two-tailed Student’s *t* tests (two-group comparisons). A value of *p* < 0.05 was considered to indicate statistical significance.

## Results

### TR4 is associated with sepsis prognosis

**Fig. 2:**
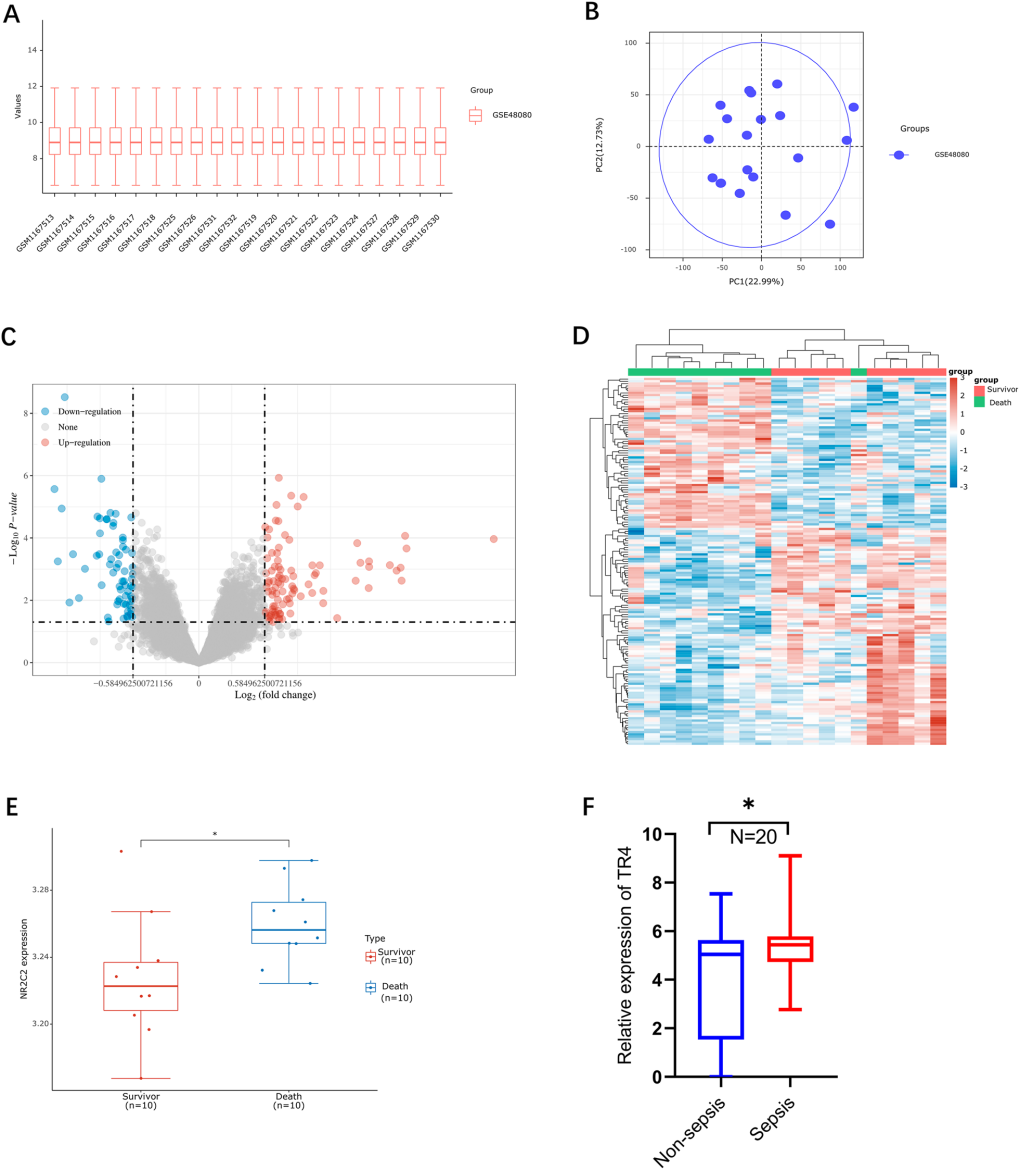
TR4 is associated with the sepsis prognosis. **A** Normalize the gene expression values of different samples by log2 transformation. **B** Principal component analysis (PCA) was performed on different samples. **C** Volcano plot of differentially expressed genes; red dots represent upregulated genes and blue dots represent downregulated genes. The fold change was set as ≥ 2 or ≤ 0.5, *p* < 0.05. **D** Heatmap of the top 50 differentially expressed genes. **E** Expression of TR4 in the sepsis survival and death groups. *N* = 10, *p < 0.05. **F** Relative mRNA expression of TR4 in the sepsis and nonsepsis groups. *N* = 20, *p < 0.05

We obtained the mRNA data set (GSE48080), which is associated with sepsis, from the GEO database, which included 10 surviving sepsis patients and 10 nonsurviving sepsis patients. We aimed to obtain the differentially expressed genes (DEGs) via the use of the ACLBI web tool (https://www.aclbi.com/). The extracted data were normalized by log2-transformation. The microarray data were normalized by the normalize quantiles function of the preprocessCore package in R software. Probes were converted to gene symbols according to the annotation information of the normalized data in the platform. Probes matching multiple genes were removed from these data sets. The average expression value of genes measured by multiple probes was calculated as the final expression value. After data standardization, we removed the batch effects using the removeBatchEffect function of the limma package in R software, because the data were from different data sets or from the same data set but on different platforms. The results of the data preprocessing were evaluated via boxplots (Fig. 2A). As shown in the schematic diagram, the PCA results showed the intersection of the data sets, which can be used as a batch of data for subsequent analysis (Fig. 2B). With the criteria *p* < 0.05 and |fold change (FC)|≥ 1.5, we identified 178 upregulated genes and 144 downregulated genes (Fig. 2C, D).

**Fig. 3:**
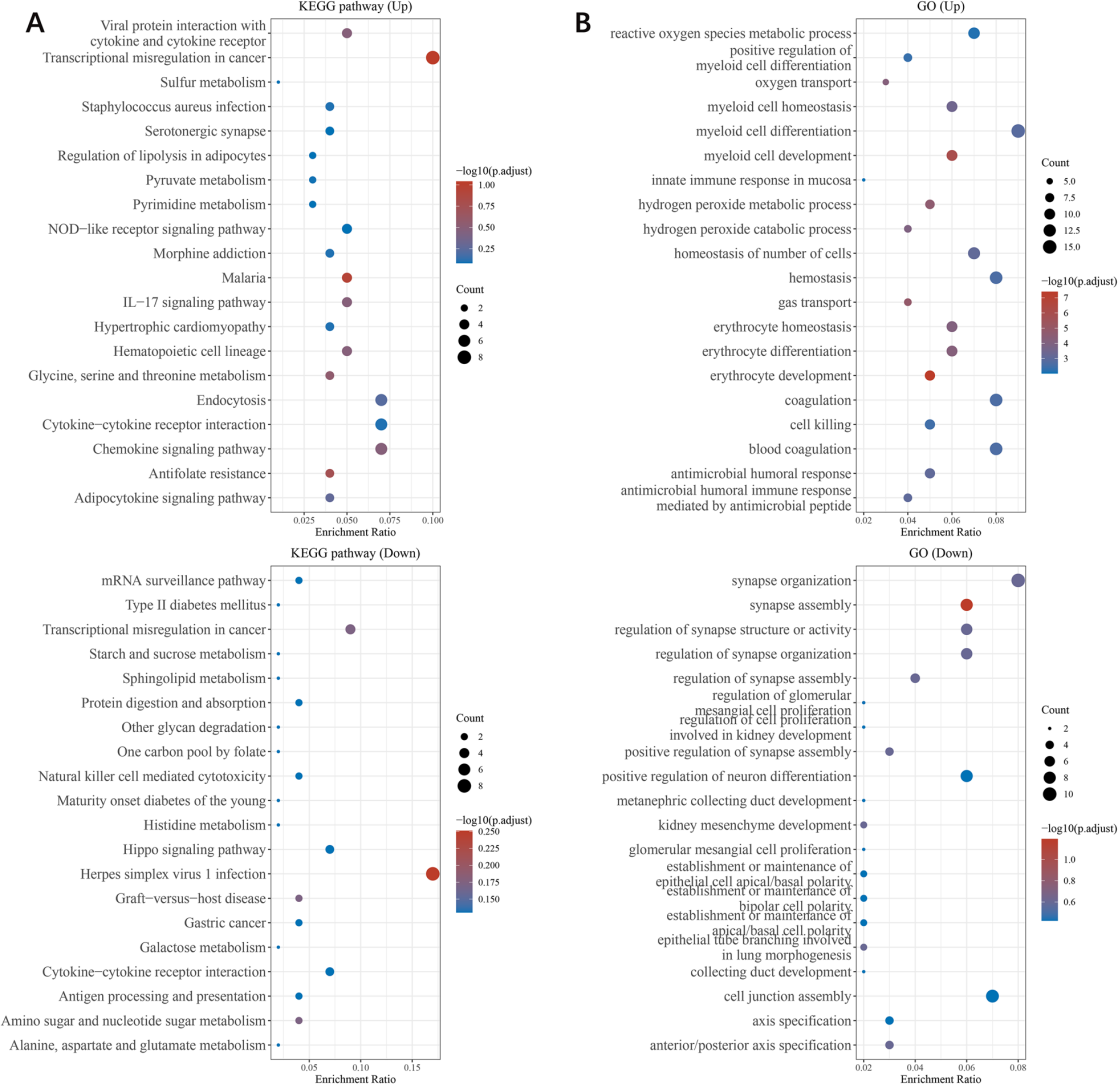
Functional analysis of DEGs. **A** Enriched KEGG signaling pathways were selected to demonstrate the primary biological actions of major potential mRNAs. The abscissa indicates the gene ratio and the enriched pathways are presented on the ordinate. **B** GO analysis of potential targets of mRNAs. The biological process (BP), cellular component (CC), and molecular function (MF) categories of the potential targets were clustered based on the ClusterProfiler package in R software (version 3.18.0). According to the enrichment results, genes with *p* < 0.05 or FDR < 0.05 were considered to be enriched in a meaningful pathway

Moreover, Gene Ontology (GO) and Kyoto Encyclopedia of Genes and Genomes (KEGG) analyses were performed to investigate the specific biological functions of these genes. The upregulated genes were involved in the key pathways linked to viral protein interactions with cytokines and cytokine receptors and transcriptional misregulation. Further GO analysis revealed that these genes were enriched in biological processes, such as reactive oxygen species metabolic process, myeloid cell homeostasis, myeloid cell differentiation and myeloid cell development. The downregulated genes were also involved in pathways linked to transcriptional misregulation and cytokine–cytokine receptor interaction. GO analysis revealed the downregulated genes were involved in the processes of synapse organization, synapse assembly, and regulation of synapse structure or activity (Fig. 3A, B). These results indicated that transcriptional regulation and myeloid cell homeostasis play important roles in the sepsis.

Among the 177 upregulated genes, the transcription factor (TF) TR4, which was the focus of our group, was associated with sepsis prognosis. TR4 expression was significantly greater in the nonsurvivor group than in the survivor group (Fig. 2E). Moreover, we also found that the TR4 mRNA expression was greater in the sepsis group than in the nonsepsis group (Fig. 2F). In summary, these results suggested that TR4 might play a crucial role in sepsis.

### TR4 worsens the outcomes of urosepsis

**Fig. 4:**
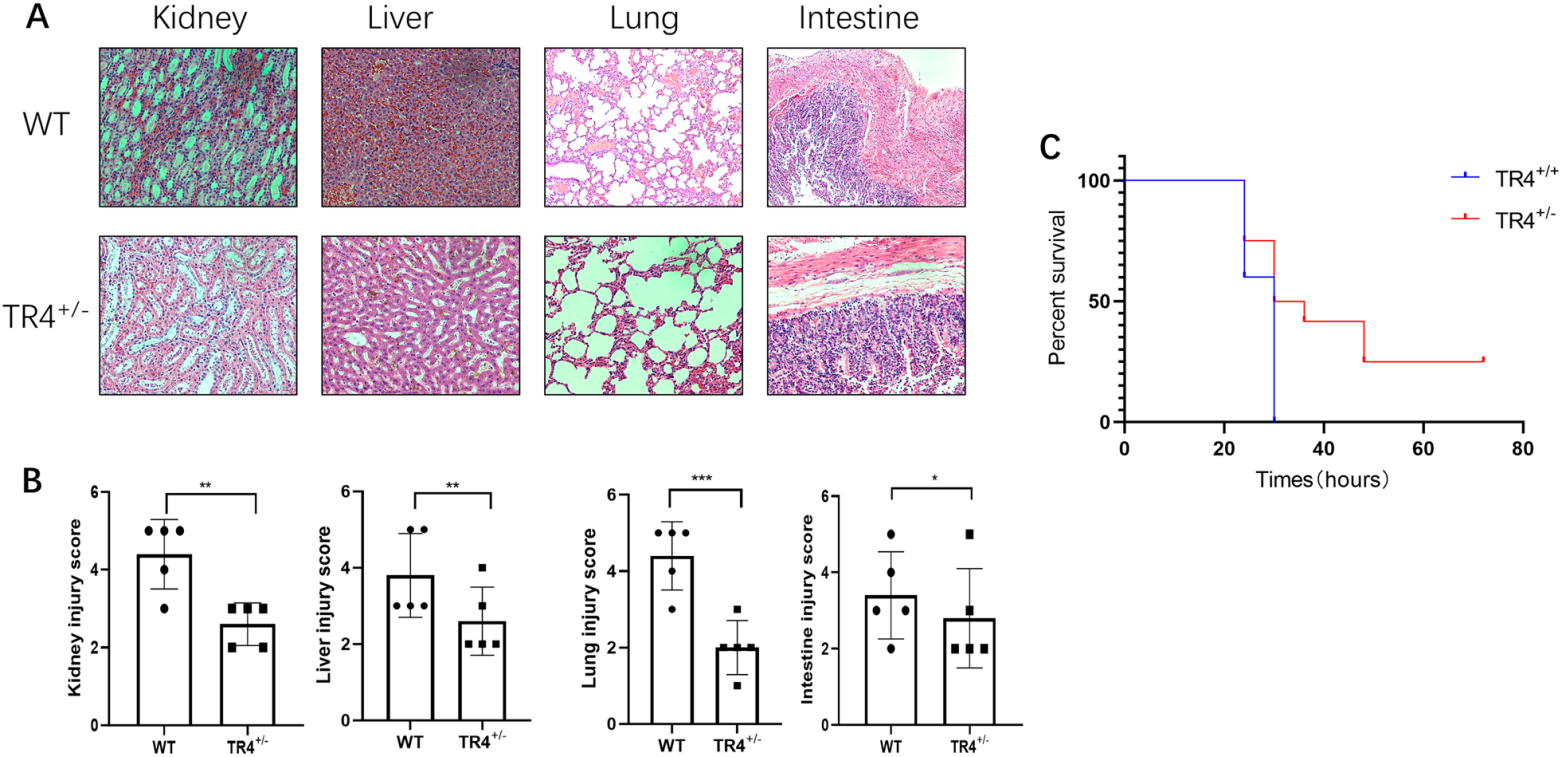
TR4 worsens the outcomes of urosepsis. **A** HE staining of the kidney, liver, lung, and intestine of TR4 knockdown mice and TR4 WT mice induced by *E. coli* bacteria. **B** Quantification of the injury score based on H&E-stained sections of WT mice and TR4^±^ mice; *N* = 5; *p < 0.05, **p < 0.01, ***p < 0.001. **C** Overall survival time of the WT mice and TR4^±^ mice; *N* = 10

To evaluate the role of TR4 in urosepsis development in vivo, we constructed TR4-knockdown mice (TR4^±^), which were generated in the previous study [30]. We injected *E.coli* bacteria into the renal pelvis of mice, a commonly used urosepsis model [38], to monitor survival and pathophysiological changes. Bacteria disseminate systemically and appear to localize to different organs, including the liver, kidneys, lungs, intestines, heart, and brain [39]. However, the liver, kidneys, lungs, and intestines are the organs most easily and frequently affected in the process of urosepsis [34, 38, 40, 41]. Moreover, due to their crucial roles in maintaining homeostasis and metabolic regulation in the body, the kidney, lung, intestine, and liver were chosen for histopathological analyses. Interestingly, histopathological analysis of the lung, liver, kidney, and intestine revealed that compared with those in the TR4-knockdown group, the organ injury in the WT group was more severe at 24 h after injecting *E. coli* (Fig. 4A, B). Furthermore, in the WT mouse group, all the mice died within 24 h, whereas in the TR4^±^ group, 25% of the mice died within 24 h, and 75% of the mice died after 72 h (Fig. 4C). These data suggest that high TR4 expression in mice leads to poor outcomes during sepsis.

### TR4 promotes pyroptosis during urosepsis

**Fig. 5:**
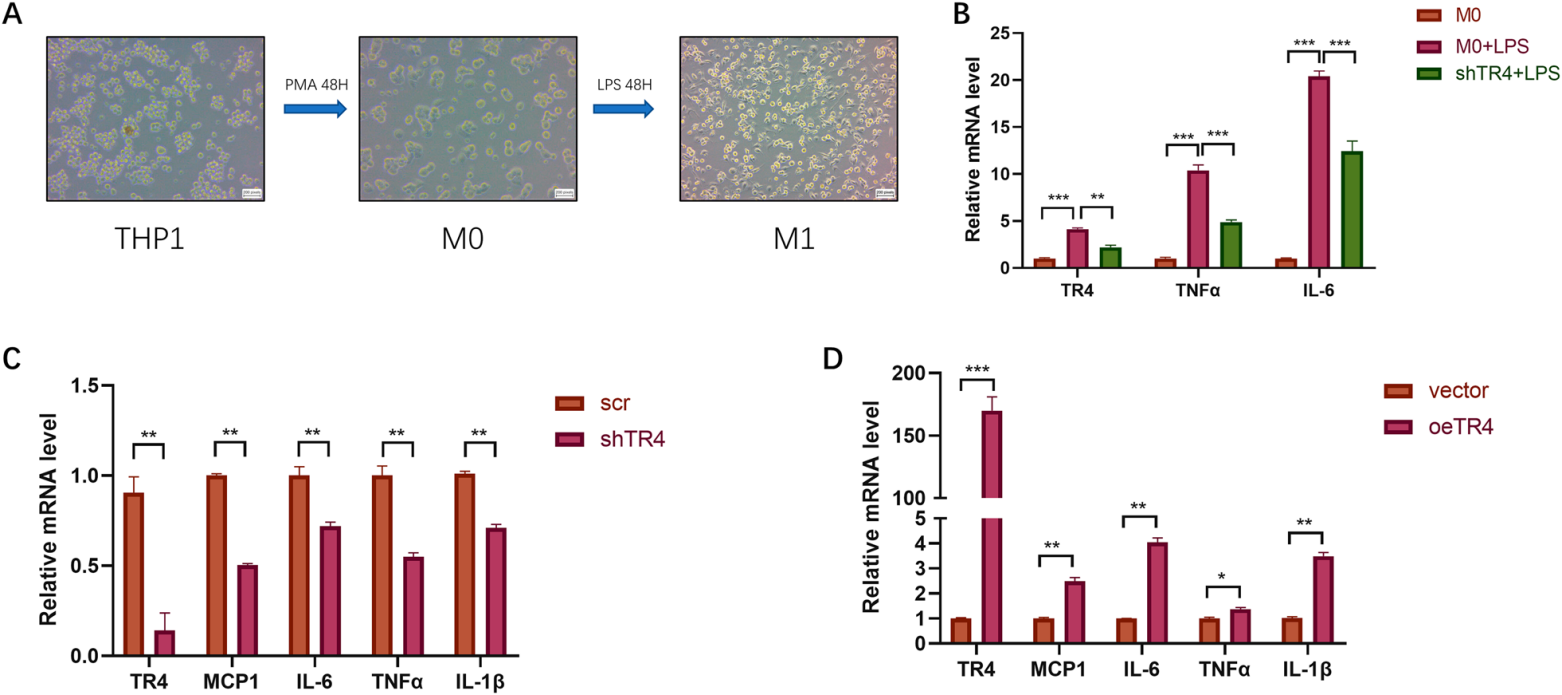
TR4 promotes inflammatory factor secretion during urosepsis. **A** THP-1 cells can be induced to differentiate into M0 macrophages after treatment with 100 ng/ml PMA for 48 h, and M0 macrophages, which are round or oval in shape, can be observed under a microscope. The M0 cells were then treated with 500 ng/ml LPS for 48 h to induce M1 macrophages. Under the microscope, irregular cell morphology and obvious cell protrusions could be observed. **B** After LPS stimulation, the expression levels of TR4, TNFα, and IL-6 in macrophages were significantly increased. When TR4 was knocked down, the expression levels of TR4, TNFα, and IL-6 in macrophages decreased. *p < 0.05, **p < 0.01, ***p < 0.001. **C** After knocking down TR4, the expression levels of MCP1, IL-6, TNFα, and IL-1β were significantly decreased; **p < 0.01. **D** After overexpressing TR4, the expression levels of MCP1, IL-6, TNFα, and IL-1β were significantly increased; *p < 0.05, **p < 0.01, ***p < 0.001

Urosepsis is a fatal condition characterized by a dysregulated host reaction to microbial infection [42]. Therefore, we questioned whether TR4 could affect the inflammatory factors. We used THP-1 cells to investigate the potential function of TR4 (Fig. 5A). After TR4 was knocked down, the expression of cytokines, such as TNF-α and IL-6, induced by LPS decreased (Fig. 5B). After overexpressing TR4 in vitro, we found that the inflammatory cytokines such as MCP1, TNF-α, IL-1β and IL-6 were significantly greater than those in the control group, whereas after knocking down TR4, the inflammatory cytokines were significantly lower (Fig. 5C, D). These results suggest that the effect of TR4 on urosepsis is attributable to a dysregulated inflammatory cytokinesis.

**Fig. 6:**
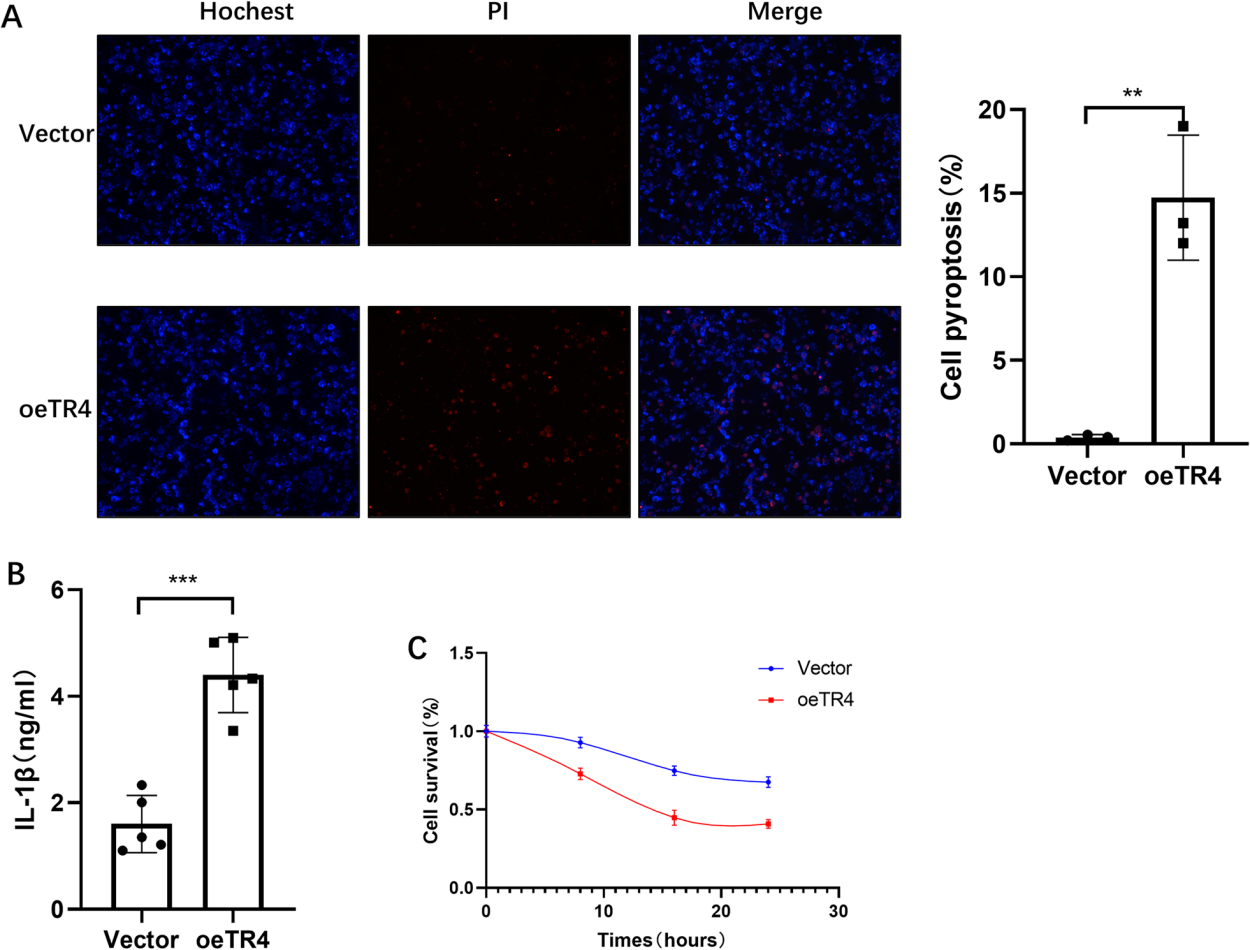
TR4 promotes pyroptosis during urosepsis. **A** Propidium iodide (PI; red indicates dead cells) and Hoechst (blue indicates all nuclei) double staining were used to observe the morphology of pyroptotic cell, and the numbers of pyroptotic cells were compared; *p < 0.05, **p < 0.01, ***p < 0.001. **B** Comparison of the IL-1β concentration in the culture supernatant of control and TR4-overexpressing macrophages; *p < 0.05, **p < 0.01, ***p < 0.001. **C** Comparison of 24-h pyroptotic cell numbers in control and TR4-overexpressing macrophages

**Fig. 7:**
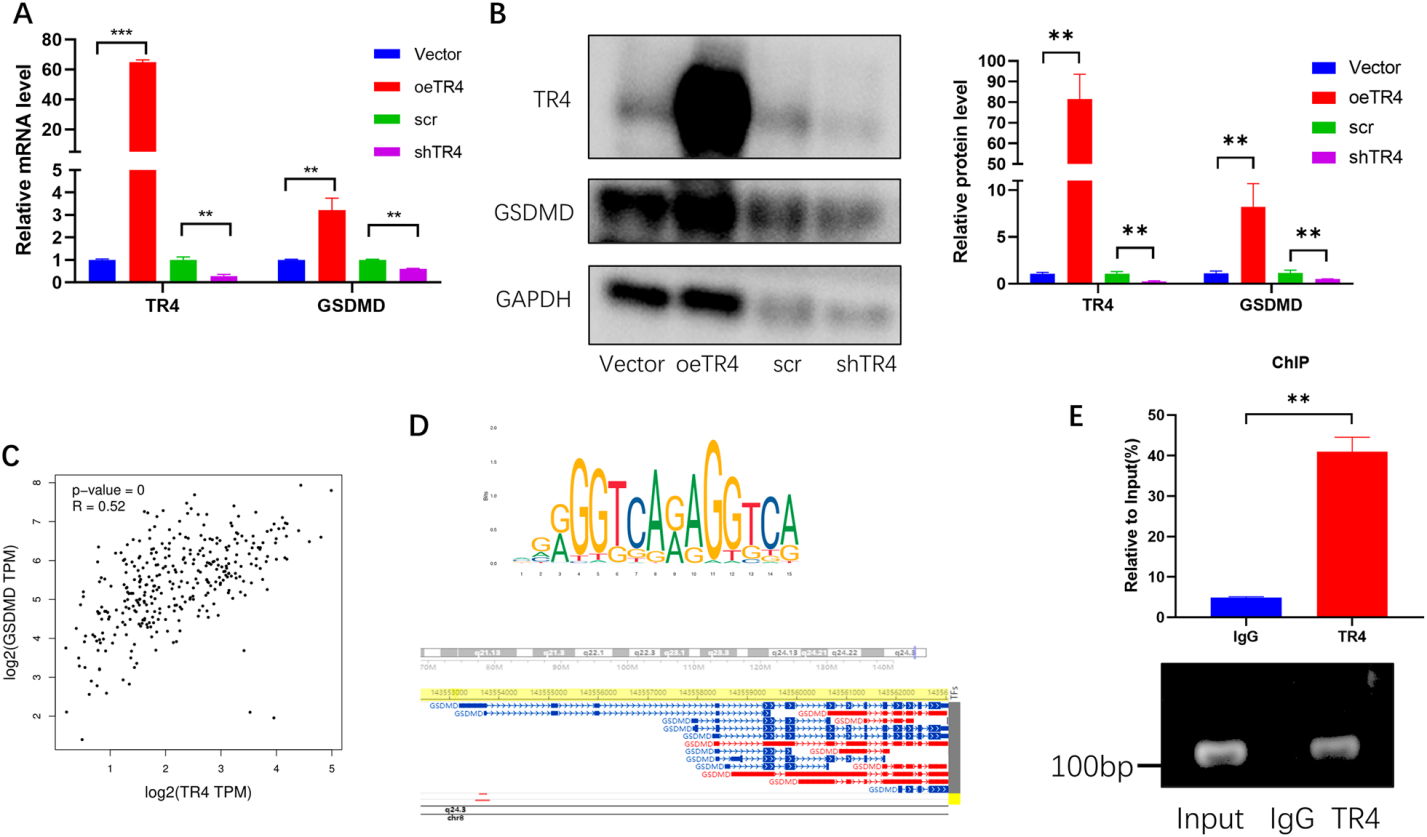
TR4 regulates the expression of GSDMD. **A** The expression of GSDMD mRNA was changed when TR4 was overexpressed or knocked down, *p < 0.05, **p < 0.01, ***p < 0.001. **B** Changes in the protein level of GSDMD when TR4 was overexpressed or knocked down. The quantification of TR4 and GSDMD expression based on band density is shown in the right panel. **C** Correlation between TR4 and GSDMD expression in blood cells. **D** The upper picture shows the motif structure of TR4 predicted by the website, and the lower picture shows the promoter region of GSDMD to which TR4 may bind, which was predicted by the hTFtarget website. **E** ChIP experiments verified that TR4 binds to the GSDMD promoter region (chr8:143553465–143,553867); **p < 0.01

Pyroptosis is a programmed cell death process related to inflammation cytokine storms [43]. We tested whether TR4 was associated with pyroptosis using PI/Hoechst staining, which was a classic method for detecting pyroptosis [44, 45].As the pyroptotic cell death was revealed by PI staining, we used this approach to assay pyroptosis. Interestingly, we found that overexpressing TR4 significantly induced more pyroptosis cells compared with the control group (Fig. 6A). We also detected the mature IL-1β levels in the culture supernatants, and found that TR4 markedly increased IL-1β secretion (Fig. 6B). Furthermore, the CCK-8 assay results also confirmed that overexpressing TR4 induced more pyroptosis (Fig. 6C). GSDMD is a key molecule in pyroptosis. GSDMD is cleaved by the Caspase-1 and Caspase-11. The N-terminal of the GSDMD forms a pore in the membrane to release cytokines [16]. Interestingly, after overexpressing TR4, GSDMD was also upregulated at both the mRNA and protein levels. In contrast, after knocking down TR4, GSDMD was also downregulated (Fig. 7A, B). To characterize the correlation between TR4 and GSDMD, we performed Spearman correlation analysis using GTEx data of the whole blood. TR4 expression was significantly positively correlation with GSDMD expression in whole blood (Spearman 0.52, *p* < 0.01; Fig. 7C). Furthermore, we used the JASPAR database to scan the GSDMD promoter region for potential TR4 response elements and found a potential binding site (chr8:143553465–143553867) present in the GSDMD promoter region (Fig. 7D). Subsequently, we performed ChIP assays and confirmed that TR4 could bind to the GSDMD promoter region (Fig. 7E).

Taken together, these results demonstrated that TR4 promoted pyroptosis by mediating the expression of GSDMD (Fig 7).

## Discussion

In this study, TR4 was upregulated in the nonsurviving group compared with surviving group. Furthermore, we confirmed that TR4 promoted the urosepsis progression through pyroptosis by upregulating GSDMD expression. These results revealed that TR4 plays an important role in urosepsis.

**Fig. 8:**
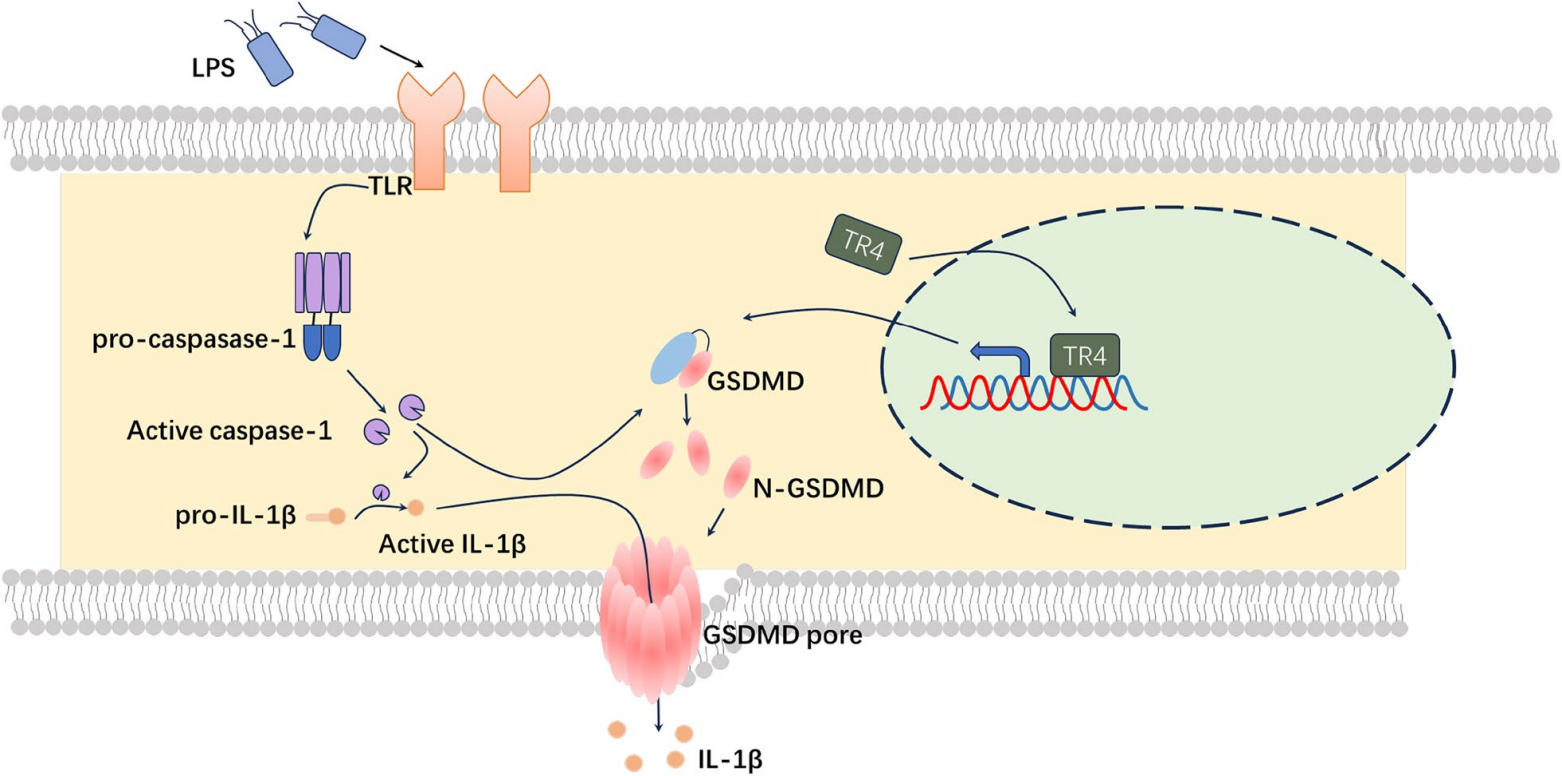
Hypothetical model by which TR4-regulated macrophage pyroptosis promotes urosepsis via GSDMD. The mechanism map of the TR4 acts as a key regulator of GSDMD gene expression and pyroptosis in macrophages

Despite advances in therapeutic interventions, urosepsis remains a substantial global burden of morbidity and mortality [46–48]. Pyroptosis is a newly identified form of programmed cell death accompanied by the release of a large number of proinflammatory factors and the induction of a cascade of amplified inflammatory responses [49]. Pyroptosis was first detected in macrophages and related diseases [50]. In recent years, pyroptosis has attracted increased amounts of attention, and increasing evidence has suggested that pyroptosis is associated with cytokine storms during sepsis [51]. GSDMD is vital for pyroptosis in mice and humans. The GSDMD protein contains both the N terminal and the C terminal structural domain, and the N terminal domain plays a major role in the induction of pyroptosis [52]. The Feng Shao group described the detailed function of GSDMD. Caspase-1, caspase-4, and caspase-11 cleave the GSDMD in response to infection. The resultant N-terminal GSDMD fragment forms the pore on the membrane that disrupts the membrane and releases IL-1β during pyroptosis [53]. GSDMD-deficient cells resisted the induction of pyroptosis by LPS, and the release of IL-1β was also impaired in the absence of GSDMD [16]. Consistent with these results, GSDMD-deficient mice had longer survival time than the WT mice during the sepsis induced by LPS. In addition, GSDMD-deficient mice released less IL-1β and LDH [54]. However, little is known about the regulation of GSDMD expression. As shown in the Nobuhiko group, IRF2, a transcription factor, directly bind to the GSDMD promoter and induces GSDMD expression. Similar to GSDMD deficient mice, IRF2 deficient mice are also protected from LPS-induced lethal septic shock [54]. In the present study, we observed that TR4 could also bind to the GSDMD promoter region and induce GSDMD expression. TR4-knockdown mice were protected from the urosepsis. These findings revealed that TR4 might be a potential therapeutic target for urosepsis (see Fig. 8).

Recently, the heterogeneity of sepsis, which results in a variety of immune responses and progresses to different conditions, has severely hindered the progression of urosepsis [55–57]. Therefore, the underlying genetic variants need to be understood for effective targeted therapies for urosepsis. A precise approach to identifying specific genes could lead to the identification of subgroups of patients with different immune responses who may benefit from personalized therapy [58–60]. In this study, we found that TR4 may be a potential biomarker for urosepsis. Notably, targeting TR4 might be a potential treatment for urosepsis patients via protection against pyroptosis. Metformin, a TR4 inhibitor [61], has been proven to protect against pyroptosis in various studies [62–64]. These results not only provide evidence that TR4 is involved in pyroptosis, but also suggest a potential medicine for the treatment of urosepsis. However, the effect of metformin on urosepsis needs additional studies.

However, this study has several limitations. The number of human sepsis samples collected is inadequate, and further clinical experiments in humans are needed. Additional studies are needed to further investigate the effect of TR4 on the detailed regulation expression of GSDMD expression. Hence, a macrophage-specific TR4 knockout mouse model should be created for future studies. Moreover, the effect of TR4 inhibitor on urosepsis should be investigated in an animal study.

Taken together, this study revealed that TR4 plays an important role in urosepsis. Targeting TR4 may lead to new therapeutic approaches for the management of urosepsis and other typical and atypical inflammasome-mediated conditions.

## Data Availability

The data that support the findings of this study are available from the corresponding author upon reasonable request.
